# Epidemiology of gastrointestinal parasites of dogs in four districts of central Ethiopia: Prevalence and risk factors

**DOI:** 10.1371/journal.pone.0316539

**Published:** 2025-01-14

**Authors:** Kibruyesfa Bayou, Getachew Terefe, Bersissa Kumsa

**Affiliations:** 1 School of Veterinary Medicine, Wollega University, Nekemte, Ethiopia; 2 Department of Pathology & Parasitology, College of Veterinary Medicine and Agriculture, Addis Ababa University, Bishoftu, Ethiopia; Van Yuzuncu Yil University Faculty of Veterinary Medicine: Yuzuncu Yil Universitesi Veteriner Fakultesi, TÜRKIYE

## Abstract

From February 2022 to April 2023, a cross-sectional study on dog gastrointestinal parasites was conducted in Bishoftu, Dukem, Addis Ababa, and Sheno, Central Ethiopia, with the aim of estimating the prevalence and evaluating risk factors. A total of 701 faecal samples were collected and processed using floatation and McMaster techniques. In dogs that were investigated, the overall prevalence of gastrointestinal parasites was 53.1% (372/701). Nematode (28.2%), cestode (8.4%), and protozoan (5.6%) parasite infections were detected in dogs in both single (42.2%) and combined (10.8%) infections. With respective prevalences of 16%, 9.8%, 5%, 3.9%, and 3.1% *Ancylostoma* spp., *Toxocara canis*, *Dipylidium caninum*, *Giardia* spp., and *Taenia/Echinococcus* spp. were the most common parasites. The prevalence of gastrointestinal parasites was significantly higher (P<0.05) in female dogs (73.8%, OR = 0.4), adult dogs (55.3%, OR = 0.4), dogs that were given raw food (57.9%, OR = 2.7), and dogs kept free outdoor (60.9%, OR = 2.4). The incidence of gastrointestinal parasites was also higher in dogs with diarrheal faecal consistency (89.1%, OR = 9.1) and dogs from highland areas (62.1%, OR = 1.8). In contrast, statistically significant variation in the prevalence of gastrointestinal parasites was not recorded among dogs of different breeds. The current study found that dogs in the studied locations had a high overall prevalence of gastrointestinal parasites. In conclusion, gastrointestinal parasites in dogs have the potential to pose a serious threat to public health, so addressing this issue requires a unified approach. Therefore, it is necessary to conduct detailed epidemiological and genetic research on dog parasites in vast study regions across various agro-ecologies zones and seasons in Ethiopia. Additionally, it is crucial to raise public awareness of the prevalence, effects on public health, and financial implications of dog gastrointestinal parasites in Ethiopia.

## 1. Introduction

With a lengthy history of coexisting with humans, the dog was the first domesticated animal [1]. Dogs provide a variety of purposes for humans, including hunting, security, police assistance, military service, companionship, and, more recently, helping persons with disabilities [2, 3]. Disease agents have spread as a result of this coexistence. These agents can infect one or more animal species, serving as important intermediate hosts for dog parasites [1].

Intestinal parasite infections are common in dogs [4]. The most prevalent organisms causing gastrointestinal disorders in dogs are parasites, mainly helminths and protozoa. These parasites cause diarrhoea, vomiting, anorexia, a dull coat, intestinal mucosal irritation, and bleeding. These can result in anaemia and possibly death [5]. *Toxocara canis*, *Ancylostoma caninum*, *Taenia hydatigena*, *Echinococcus* species, *Dipylidium caninum*, *Trichuris vulpis*, *Giardia* species, *Cryptosporidium* species, and *Cystoisospora* species are the most prevalent intestinal parasites in dogs [6].

Dogs living close to people are typically seen to be a major contributing element to the spread of zoonotic diseases, which can have detrimental effects. *Strongyloides stercoralis*, *Ancylostoma caninum*, *Dipylidium caninum*, *Toxocara canis*, *Echinococcus granulosus*, and *Trichuris vulpis* are the most prevalent zoonotic helminth parasites in dogs [7]. The oral-faecal cycle is how canine intestinal parasites spread. The release of eggs or larvae and oocysts or cysts into the environment is the main way that these parasites propagate. Direct contact with the definitive host, contaminated water and food, or indirect interaction with animal secretions and excreta can all spread zoonotic agents [6].

The prevalence of endoparasites in dogs varies from 5% to 70% in various regions [8–11]. According to these investigations, the most prevalent intestinal parasites in dogs are *Trichuris* species, *Toxocara* species, *Ancylostoma* species, and *Cystoisospora* species [12]. Therefore, ongoing research on the prevalence of various dog gastrointestinal parasites (GIT) and associated risk factors is essential for effective parasite control [13]. Dogs have a significant role in both urban and rural Ethiopian families, often acting as companion animals and house guardians [14]. There is a lack of reliable information on Ethiopia’s dog and cat numbers [15]. Nonetheless, it is estimated that there are more than 5 million dogs in the nation [16]. The majority of Ethiopian dogs are stray animals that wander the streets, homesteads, slaughterhouses, butcher shops, and marketplaces. It has been discovered that these dogs have the largest parasite burdens [17, 18]. There are a lot of strays and unconfined dogs with a high prevalence of zoonotic parasites that pollute the environment, which is very concerning given the lack of public awareness about the welfare of these animals and the risks to public health from their diseases [18].

The prevalence of canine intestinal parasites in Ethiopia has been reported in a limited number of studies [19–21] with gastrointestinal helminth prevalence in dogs ranging from 52.9% to 94.6% [13, 22–24]. There are, however, few recent data on the incidence of canine gastrointestinal helminth parasites from central Ethiopia, specifically from Bishoftu, Dukem, Addis Ababa, and Sheno.

Information on gastrointestinal parasites that affect dogs in Ethiopia need therefore to be updated and expanded. It is essential to comprehend the epidemiology and all other facets of the disease in order to develop a practically relevant control program against diseases of high public health concern [25]. In order to establish baseline data for future initiatives aimed at reducing the risks to the human population, the study’s goals were to estimate the prevalence of various gastrointestinal parasites in dogs and evaluate the associated risk factors in the Central Ethiopian districts of Bishoftu, Dukem, Addis Ababa, and Sheno.

## 2. Materials and methods

### 2.1. Study areas

The first research area, Bishoftu town (Ada’a district), is located 47 km southeast of Addis Ababa at an elevation of 1850 meters above sea level, with latitudes 9°N and longitudes 40°E. The area has a bimodal rainfall pattern, with a longer wet season from June to September and a shorter rainy season from March to May. It receives 866 mm of rainfall annually, of which 84% falls during the long rainy season and the remainder during the short one. October through February is considered the dry season. The region experiences 61.3% relative humidity and average annual maximum and minimum temperatures of 26°C and 14°C, respectively [26]. According to species, there are 160,697 cattle, 22,181 sheep, 37,510 goats, 1660 horses, and 191,380 poultry [27], while the estimated number of dogs is 4188 [28].

Dukem, the second research location, is located adjacent to the main road to Adama, 37 km southeast of Addis Ababa. Between latitudes 8045’25" N and 8050’30" N and longitudes 38051’55" E and 38056’5" E, the study area physically covers 9,630.6 hectares. On average, it is about 2,100 meters above sea level. The average yearly high and low temperatures in the region are 27°C and 22°C, respectively and with an annual rainfall of *95 mm*. The towns of Bishoftu and Gelan, which are located in the southeast and the majority of the north, respectively [29]. It is believed that there are 944 dogs in Dukem [28].

Addis Ababa, the third research area, is located 2408 meters above sea level and at latitudes 90 3’ N and 380 43’ E. The average annual rainfall is 1201 mm, and the average lowest and highest temperatures are 9.4 and 23.2°C, respectively. Rainfall has a bimodal trend, with the months of February through April seeing the smallest showers and June through September seeing the longest and heaviest rainfall [30]. Between 250,000 and 350,000 dogs are thought to live in Addis Ababa, with half of them being owned [31].

Sheno, the fourth research location, is situated in the North Showa Zone and is 78 kilometres from Addis Ababa, the country’s capital. Sheno is situated between 1950 and 2918 meters above sea level at latitude 9°20’N and longitude 39°18’E. The terrain is flat and the agro-ecology is highlands. Bimodal rainfall patterns with unpredictable distribution are what define the region. Rainfall averages 1366.7 mm per year. The average yearly high and low temperatures in the region are 19.9°C and 12.9°C, respectively. Dogs are thought to number 4188, while the area’s livestock population consists of 1.51 million cattle, 1 million sheep, 223,245 goats, donkeys, horses, and mules, with respective numbers of 254,553, 107,368 and 3,739 [32].

### 2.2. Study design

From February 2022 to April 2023, a cross-sectional study was carried out to determine the prevalence of gastrointestinal parasites in dogs and evaluate the associated risk factors (like origin, sex, age, breed, feeding condition, housing condition, agro-ecology and faecal consistency).

### 2.3. Sample size and sampling technique

The formula [33] for random sampling was used to get the necessary sample size. Based on an anticipated prevalence rate of 59.2% for dog gastrointestinal parasites, the sample size was determined [23]. Consequently, the following formula was used to determine the sample size:

$$n=\frac{1.96^{2}\;Pexp(1‐Pexp)}{d^{2}}$$

Where: n = desired sample size, Pexp = expected prevalence, and d = 0.05

According to the calculations, 371 dogs were anticipated to be part of the study; nevertheless, in order to improve the accuracy of the study’s findings, 701 dogs (Bishoftu (305), Dukem (135), Addis Ababa (134) and Sheno (127)) were specifically taken into consideration for faecal and data collection in this study.

### 2.4. Sample collection

A verbal consent was informed to the dog owners and with their cooperation faecal samples were collected using disposable gloves. From each study animal 5–10 grams faecal sample was collected either straight from the rectum or from the top layer of recently voided faeces [24]. Then, the collected faecal samples were placed in labelled clean plastic containers (universal bottles) and preserved in 10% formalin then transported in icebox to the College of Veterinary Medicine and Agriculture of Adds Ababa University parasitology laboratory for further processing on the same day of collection. Sample left unprocessed on the same day were examined on the following days. A data recording format was used to document the study site, animal breed, sex, age, food and housing conditions, and the consistency of their faeces at the time of sampling. Physical examination of the faeces during sample collection was used to assess the consistency of the faeces. Dogs with the age < 6 months, 6 months up to 1 year and ≥ 1 year were considered as puppy, young and adult dogs, respectively [34].

### 2.5. Parasitological procedures

To find gastrointestinal parasite eggs or cysts, faecal samples were analyzed using the centrifuge-flotation technique in a saturated zinc sulphate solution (specific gravity = 1.20) [35]. Hence, around three grams of the faecal sample were weighed and put into mortar. Ten millilitres of flotation fluid (zinc sulphate solution) were then added, and the faecal sample inside the mortar was completely combined and crushed with a pestle. To get rid of the coarse debris, the solution was sieved into a beaker using a tea strainer. After adding the filtrate to the centrifuge tube and centrifuging it for three minutes at 3000 rpm, the floatation fluid was added until a cone-shaped top formed. A coverslip was then placed on top of the tube and let to stand for fifteen to twenty minutes. The coverslip was carefully lifted up and put on the microscopic slide [35, 36].

The entire slide was examined under a microscope, and parasite eggs, trophozoites, and cysts were discovered using a compound microscope with a 10x objective lens and a 40x objective magnification. To make it easier to identify protozoa and cysts, iodine solution was utilized. Morphologically, parasites were identified to the level of genera or species using ova/cyst identification keys, which were provided by [37, 38]. When at least one kind of parasite egg or cyst was found, the sample was deemed positive [39]. The McMaster technique was then used to re-analyse faecal samples that tested positive for helminth and protozoan ova using the flotation technique in order to assess the intensity/level of infection of each parasite in the study areas.

The number of eggs, cysts/oocysts per gram of faeces was calculated based on [40] as follows: three grams of faeces were mixed in a clean glass beaker with 42 ml of flotation solution (zinc sulphate solution) until the mixture is homogeneous, the mixture filtered with a sieve and the filtrate collected in a new beaker. The filtrate was taken with a pipette to fill both chambers of McMaster slide and let the slide stand for 5 minutes to allow parasite eggs/cysts to float to the surface. Then all eggs/cysts inside of the grid areas of both chambers were counted under microscope. Then, the total eggs/cysts per gram (EPG) of faeces was calculated. The total number of egg, cyst/oocysts of the two chambers was multipled by 50. This gives the EPG of faeces. Then based on [40], the EPG scores (intensity of infection) were categorized into three groups: mild, EPG < 1,000; moderate, 1,000 ≤ EPG < 10,000; severe, EPG ≥ 10,000.

### 2.6. Ethical consideration

The Addis Ababa University, College of Veterinary Medicine and Agriculture Research Ethics Committee (AAU-CVMA-REC) and the animal welfare guide for the care and use of animals (Ref. No.VM/ERC/36/02/15/2023) approved the animal handling ethics.

### 2.7. Data management and analysis

Microsoft Excel sheet 2010 was used to enter all of the generated data. IBM SPSS version 27, the Statistical Package for Social Sciences, was used to analyze the data. The relationship between helminths and protozoan parasitism and the risk factors (research site, sex, age, breed, feeding and housing conditions, faecal consistency, and agro-ecology) was examined using descriptive statistics and the chi-square (χ2) test. Binary logistic regression was used to compute the odds ratio (OR) and confidence interval for each risk factor, using the category with the lowest prevalence as a baseline for comparison. ANOVA was utilized to compare the means of three or more groups in order to analyze continuous data (faecal egg count or egg per gram of faeces (epg)). The statistical significance threshold was set at a P-value of less than 0.05.

## 3. Results

### 3.1. Overall prevalence of gastrointestinal parasites in dogs

**Fig 1:**
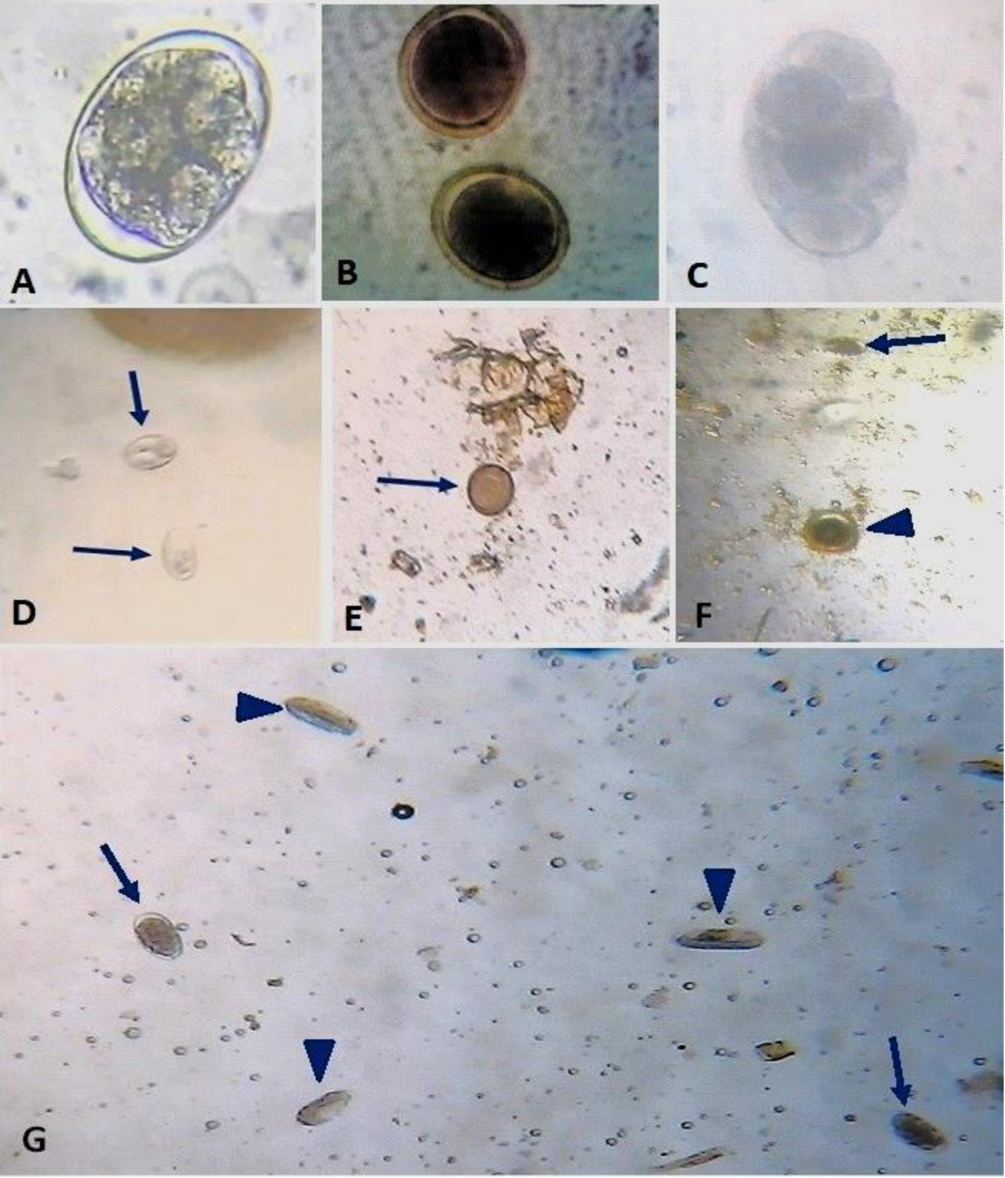
*Ancylostoma* spp. from 2 yrs. female dog X40 (A), *Toxocara canis* of 1.5 yrs. male dog X40 (B) from Bishoftu, *Dipylidium caninum* of 4 yrs. old male dog X40 from Addis Ababa (C). *Giardia* spp. from 4 yrs. male dog X10 (A) from Dukem (D). *Taenia* spp. from 3 yrs. male dog X10 from Sheno (E). Mixed infection of *Ancylostoma* spp. (arrow) and *Toxocara canis* (arrowhead) from 4 yrs. male dog X10 from Dukem (F). Mixed infection of *Ancylostoma* spp. (arrow) and *Physaloptera* spp. (arrowhead) from 2yrs. female dog X10 from Bishoftu (G).

Using coproscopic techniques, 701 dogs from Bishoftu, Dukem, Addis Ababa, and Sheno were examined for any gastrointestinal parasites. In all, 372 dogs (53.1%) tested positive for at least one kind of gastrointestinal parasite (Fig 1).

Bishoftu had a significantly (P<0.001) lower prevalence of gastrointestinal (GI) parasites than the other study locations. The prevalence of gastrointestinal parasites was significantly greater in female dogs (P<0.001) and adult dogs (P = 0.006). The frequency of GI parasites was significantly (P<0.001) greater in dogs kept free outdoors than in dogs maintained under better handling conditions. Compared to dogs that were fed cooked food, dogs that frequently consumed raw animal products were more likely to get parasite infections (P<0.001). For dogs that had previously been fed raw food, the odds ratio (OR) of gastrointestinal parasite presence was around three times greater. The prevalence of GI parasites was significantly (P<0.001) greater in dogs with diarrheal faecal consistency than in dogs with wet and dry faecal consistency. When comparing faecal samples with diarrheal consistency to samples with dry consistency, the OR of gastrointestinal parasite prevalence was around nine times greater for the former. The prevalence of GI parasites was significantly (P<0.001) greater in dogs from highland areas (Addis Ababa and Sheno) than in dogs from midland areas (Bishoftu and Dukem) (Table 1).

**Table 1:** Frequency, prevalence, 95% confidence interval and Odds ratio of gastrointestinal parasites identified in dogs relative to risk factors.

Risk factors	Category level	Frequency (%)	Number of Positive (%)	95% CI	AOR	95% CI Per Adjusted Odds Ratio		χ2 (P-value)
						Lower	Upper	
**Origin**	Bishoftu	305 (43.5)	123 (41.6)	35–46	Ref	Ref	-	35.384 (<0.001)
	Dukem	135 (19.3)	87 (64.4)	56–73	2.682	1.762	4.082	
	Addis Ababa	134 (19.1)	83 (61.9)	54–70	2.408	1.587	3.654	
	Sheno	127 (18.1)	79 (62.2)	54–71	2.435	1.591	3.727	
**Sex**	Male	617 (88)	310 (50.2)	46–54	Ref	Ref		16.487 (<0.001)
	Female	84 (12)	62 (73.8)	64–83	0.358	0.215	0.597	
**Age**	Puppy	36 (5.1)	14 (38.9)	22–56	Ref			10.109 (0.006)
	Young	48 (6.8)	17 (35.4)	21–49	0.515	0.259	1.025	
	Adult	617 (88)	341 (55.3)	51–59	0.444	0.241	0.819	
**Breed**	Cross	29 (4.1)	13 (44.8)	26–64	Ref	Ref		5.120 (0.077)
	Local	603 (86)	314 (52.1)	48–56	0.748	0.354	1.582	
	Exotic	69 (9.8)	45 (65.2)	54–77	1.726	1.025	2.904	
**Feeding condition**	Cooked	141 (20.1)	48 (34.0)	26–42	Ref	Ref		25.649 (<0.001)
	Uncooked	560 (79.9)	324 (57.9)	54–62	2.660	1.807	3.915	
**Housing condition**	Free in home/compound	175	68 (38.9)	25–41	Ref	Ref	-	40.999 (<0.001)
	Kennel/leashed	76	30 (39.5)	28–51	0.750	0.420	1.340	
	Free outdoor	450	274 (60.9)	56–65	2.387	1.452	3.925	
**Agro-ecology**	Midland	440 (62.8)	210 (47.7)	43–52	Ref	Ref		13.529 (<0.001)
	Highland	261 (37.2)	162 (62.1)	56–68	1.792	1.312	2.449	
**Faecal consistency**	Dry	56 (8)	18 (32.1)	20–45	Ref	Ref		79.195 (<0.001)
	Diarrheic	119 (17)	106 (89.1)	83–95	9.140	5.012	16.669	
	Wet	526 (75)	248 (47.2)	43–51	0.531	0.295	0.954	
**Total**		701 (100)	372 (53.1)	49–57	-	-	-	696.997 (<0.001)

95% CI, 95% confidence interval; AOR, Adjusted odds ratio, χ2, chi-square.

Single parasite infections had a significantly (P<0.001) greater prevalence of gastrointestinal parasites than mixed parasite infections (Table 2).

**Table 2:** Gastrointestinal parasite diversity of dogs examined from the four study sites.

Infection with	Origin				Total Frequency (%)	Prevalence (%)	P-value
	Bishoftu (n = 305)	Dukem (n = 135)	Addis Ababa (n = 134)	Sheno (n = 127)			
	Frequency (%)	Frequency (%)	Frequency (%)	Frequency (%)			
**No parasite**	182 (58.4)	48 (35.6)	51 (38.1)	49 (38.6)	328 (46.9)	46.9	<0.001
**One parasite**	99 (32.5)	71 (52.6)	67 (50)	58 (45.7)	296 (42.2)	53.1	
**Two parasites**	15 (4.9)	10 (7.4)	11 (8.2)	14 (11)	50 (7.1)		
**Three parasites**	9 (3)	6 (4.4)	5 (3.7)	6 (4.7)	26 (3.7)		

The incidence of nematodes was significantly (P<0.001) higher than that of protozoans and cestodes (Table 3).

**Table 3:** Group of gastrointestinal parasites detected from the faeces of examined dogs.

Type of parasite	Origin				Prevalence (%)	Total Prevalence (%)	P-value
	Bishoftu (n = 305)	Dukem (n = 135)	Addis Ababa (n = 134)	Sheno (n = 127)			
	Frequency (%)	Frequency (%)	Frequency (%)	Frequency (%)			
**No parasite (n = 328)**	182 (55.5)	48 (14.6)	50 (15.2)	48 (14.6)	46.9	46.9	<0.001*
**Nematode (n = 198)**	69 (34.8)	44 (22.2)	45 (22.7)	40 (20.2)	28.2	53.1	
**Cestode (n = 59)**	20 (33.9)	16 (27.1)	12 (20.3)	11 (18.6)	8.4		
**Protozoa (n = 39)**	11 (28.2)	11 (28.2)	9 (23)	8 (20.5)	5.6		
**Mixed infection (n = 77)**	23 (30)	16 (20.8)	18 (23.4)	20 (25.9)	11		
**Total (n = 701)**	305 (100)	135(100)	134(100)	127(100)	100	100	

### 3.2. Prevalence of gastrointestinal parasites by origin of dogs and parasite egg densities

In the study locations, *Strongyloides stercoralis*, *Physaloptera* spp., and *Diphyllobothrium* spp. were the least common gastrointestinal parasites, while *Ancylostoma* spp. was the most common. The incidence of particular gastrointestinal parasites in dogs from the four study sites varied statistically significantly (P = 0.021) (Table 4).

**Table 4:** Frequencies and percentages of specific gastrointestinal parasites of dog.

Parasite	Bishoftu (n = 305)		Dukem (n = 135)		Addis Ababa (n = 134)		Sheno (n = 127)		Total Frequency (%)	P-value
	*N* (%)	95% CI	*N* (%)	95% CI	*N* (%)	95% CI	*N* (%)	95% CI		
**Single Parasite Infection**										
***Nematode***										
***Ancylostoma* spp.**	39 (12.8)	9–17	26 (19.3)	13–26	23 (17.2)	11–24	24 (18.9)	12–26	112 (16)	<0.001
***Toxocara canis***	21 (6.9)	4–10	16 (11.9)	6–18	20 (14.9)	9–21	12 (9.5)	4–15	69 (9.8)	<0.001
***Strongyloides stercoralis***	6 (1.9)	0–4	1 (0.7)	1–2	1 (0.8)	1–2	1 (0.8)	1–2	9 (1.3)	0.003
***Physaloptera* spp.**	4 (1.3)	0–3	2 (1.5)	1–4	1 (0.8)	1–2	2 (1.6)	1–4	9 (1.3)	0.014
***Cestode***										
***Dipylidium caninum***	12 (3.9)	2–6	9 (6.7)	2–11	7 (5.2)	1–9	7 (5.5)	1–10	35 (5)	<0.001
***Taenia/Echinococcus* spp.**	7 (2.3)	1–4	5 (3.7)	0–7	5 (3.8)	0–7	5 (3.9)	1–7	22 (3.1)	0.001
***Diphyllobothrium* spp.**	3 (0.9)	0–2	2 (1.5)	1–4	2 (1.5)	1–4	2 (1.6)	1–4	9 (1.3)	0.034
***Protozoa***										
***Giardia* spp.**	7 (2.3)	1–4	8 (5.9)	1–9	7 (5.2)	1–8	5 (3.9)	1–7	27 (3.9)	0.001
***Isospora* spp.**	4 (1.3)	0–3	3 (2.2)	0–5	2 (1.5)	1–4	3 (2.4)	0–5	12 (1.7)	0.014
**Multiple Parasite Infection**										
***Ancylostoma* spp. *and Toxocara canis***	5 (1.6)	0–3	5 (3.8)	0–7	4 (2.9)	0–6	4 (3.2)	0–6	18 (2.6)	0.006
***Ancylostoma* spp. *and Physaloptera* spp.**	3 (0.9)	0–2	1 (0.7)	1–2	1 (0.8)	1–2	1 (0.8)	1–2	6 (0.9)	0.034
***Toxocara canis and Physaloptera* spp.**	3 (0.9)	0–2	1 (0.7)	1–2	1 (0.8)	1–2	4 (3.2)	0–6	9 (1.3)	0.034
***Ancylostoma* spp. *and Taenia* spp.**	3 (0.9)	0–2	2 (1.5)	1–4	3 (2.2)	0–5	3 (2.4)	0–5	11 (1.6)	0.034
***Toxocara* spp. *and Strongyloides* spp.**	1 (0.3)	0–1	1 (0.7)	1–2	2 (1.5)	1–4	2 (1.6)	1–4	6 (0.9)	0.223
***Ancylostoma* spp., *Toxocara* spp. *and Physaloptera* spp.**	4 (1.3)	0–3	4 (2.9)	0–6	3(2.2)	0–5	3 (2.4)	0–5	14 (2)	0.014
***Ancylostoma* spp., *Toxocara* spp. *and Taenia* spp.**	2 (0.7)	0–2	1 (0.7)	1–2	2 (1.5)	1–4	1 (0.8)	1–2	6 (0.9)	0.084
***Toxocara* spp. *Physaloptera* spp. *and Giardia* spp.**	3 (0.9)	0–2	1 (0.7)	1–2	-	-	2 (1.6)	1–4	6 (0.9)	0.034
**Total**	127 (41.6)	35–46	87 (64.4)	56–73	83 (61.9)	54–70	79 (62.2)	54–71	372 (53.1)	0.021

95% CI, 95% confidence interval; *N*, number.

The parasite egg densities were calculated using the total mean egg per gram (EPG) of faeces, which came out to be 1176.8 (Table 5).

**Table 5:** Mean egg per gram of faeces (EPG) of specific gastrointestinal parasites of dog.

Parasite	Number (%) of Dogs infected	EPG		
		Mean	SD	P-value
**Single Parasite Infection**				
***Nematode***				
***Ancylostoma* spp.**	112 (16)	979**	1205	
***Toxocara canis***	69 (9.8)	1479.1***	1477	
***Strongyloides stercoralis***	9 (1.3)	761.1**	918.8	
***Physaloptera* spp.**	9 (1.3)	1444.4***	1863.8	
**Total (nematode)**	198 (28.2)	1461.1***	1339.7	<0.001
***Cestode***				
***Dipylidium caninum***	35 (5)	1265.7***	1367.4	
***Taenia/Echinococcus* spp.**	17 (2.4)	1175***	1246	
***Diphyllobothrium* spp.**	9 (1.3)	966.7**	903.3	
**Total (cestode)**	59 (8.7)	2100***	2404.1	<0.001
***Protozoa***				
***Giardia* spp.**	27 (3.9)	964.8**	1175.9	
***Isospora* spp.**	12 (1.7)	1195.8***	1365	
**Total (protozoa)**	39 (5.6)	4100***	-	<0.001
**Multiple Parasite Infection**				
***Ancylostoma* spp. and *Toxocara canis***	18 (2.6)	1233.3***	1369	
***Ancylostoma* spp. and *Physaloptera* spp.**	6 (0.9)	408.3**	156.3	
***Toxocara canis* and *Physaloptera* spp.**	9 (1.3)	1583.3***	1586	
***Ancylostoma* spp. *and Taenia/Echinococcus* spp.**	11 (1.6)	654.6**	843.7	
***Toxocara* spp. and *Strongyloides* spp.**	6 (0.9)	941.7**	770	
***Ancylostoma* spp., *Toxocara* spp. and *Physaloptera* spp.**	14 (2)	860.7**	1227	
***Ancylostoma* spp., *Toxocara* spp. and *Taenia/Echinococcus* spp.**	6 (0.9)	1925***	2048	
***Toxocara* spp. *Physaloptera* spp. and *Giardia* spp.**	6 (0.9)	2166.7***	1440	
**Total (mixed infection)**	77 (11)	1770***	1974.7	<0.001
**Total**	372 (53.1)	1176.8***	1533	0.008

SD, Standard deviation

**, mild, EPG <1,000

***, moderate, 1,000 ≤ EPG < 10,000.

### 3.3. Prevalence of specific gastrointestinal parasites by sex and age of dogs

*Ancylostoma* spp. and *Toxocara canis* eggs were the most prevalent gastrointestinal parasites in male and female dogs, respectively. Similarly, adult and young dogs were frequently found to have eggs of *Ancylostoma* spp., while puppies were primarily affected by *Toxocara canis* (Table 6).

**Table 6:** Prevalence of specific gastrointestinal parasites of dogs relative to their sex and age.

Parasite	Sex		P-value	Age			P-value
	Male (n = 617)	Female (n = 84)		Puppy (n = 36)	Young (n = 48)	Adult (n = 617)	
	*N (%)*	*N (%)*		*N (%)*	*N (%)*	*N (%)*	
***Nematode***							
***Ancylostoma* spp.**	98 (15.9)	14 (16.7)	0.783	0 (0.0)	7 (14.6)	105 (17)	0.024
***Toxocara canis***	46 (7.5)	23 (27.4)	<0.001	6 (16.7)	4 (8.3)	59 (9.6)	0.356
***Strongyloides stercoralis***	7 (1.1)	2 (2.4)	0.341	0 (0.0)	0 (0.0)	9 (1.5)	0.538
***Physaloptera* spp.**	9 (1.5)	0 (0.0)	0.265	0 (0.0)	0 (0.0)	9 (1.5)	0.538
***Cestode***							
***Dipylidium caninum***	33 (5.3)	2 (2.4)	0.241	2 (5.6)	1 (2.1)	32 (5.2)	0.628
***Taenia/Echinococcus* spp.**	14 (2.2)	8 (9.5)	<0.001	4 (11.1)	0 (0.0)	18 (2.9)	0.010
***Diphyllobothrium* spp.**	5 (0.8)	4 (4.8)	0.003	0 (0.0)	0 (0.0)	9 (1.5)	0.538
***Protozoa***							
***Giardia* spp.**	24 (3.9)	3 (3.6)	0.887	2 (5.6)	0 (0.0)	25 (4.1)	0.321
***Isospora* spp.**	12 (1.9)	0 (0.0)	0.197	0 (0.0)	0 (0.0)	12 (1.9)	0.436
**Total**	248 (40.2)	56 (66.7)	<0.001	14 (38.9)	12 (25)	278 (45.1)	0.006

N, number.

### 3.4. Prevalence of group of gastrointestinal parasites by feeding and housing conditions of dogs

The current investigation showed that dogs with uncooked food feeding experience had greater prevalences of protozoa and cestodes, while dogs with prepared food feeding experience had higher prevalences of mixed parasite infection in the study locations (Table 7).

**Table 7:** Prevalence of group of gastrointestinal parasites of dog relative to their feeding condition.

Feeding condition	Group of parasites					P-value
	No parasite	Nematode	Cestode	Protozoa	Mixed infection	
	*N (%)*	*N (%)*	*N (%)*	*N (%)*	*N (%)*	
**Cooked (n = 141)**	91 (64.5)	23 (16.3)	6 (4.3)	3 (2.1)	18 (12.8)	<0.001
**Uncooked (n = 560)**	237 (42.3)	175 (31.3)	53 (9.5)	36 (6.4)	59 (10.5)	

*N*, number.

Dogs kept free outdoor had higher rates of nematode, cestode, and protozoan infections based on housing conditions (Table 8).

**Table 8:** Prevalence of group of gastrointestinal parasites of dog relative to their housing condition.

Housing condition	Group of parasites					P-value
	No parasite	Nematode	Cestode	Protozoa	Mixed infection	
	*N (%)*	*N (%)*	*N (%)*	*N (%)*	*N (%)*	
**Kennel/leashed (n = 76)**	46 (60.5)	18 (23.7)	6 (7.9)	0 (0.0)	6 (7.9)	<0.001
**Free in home/compound (n = 175)**	105 (60)	35 (20)	9 (5.1)	10 (5.7)	16 (9.1)	
**Free outdoor (n = 450)**	177 (39.3)	145 (32.2)	44 (9.8)	29 (6.4)	55 (12.2)	
**Total**	328 (46.8)	198 (28.2)	59 (8.4)	39 (5.6)	77 (11)	

*N*, number.

## 4. Discussion

A total of 701 faecal samples were randomly selected for this cross-sectional investigation on dog gastrointestinal parasites, and they were processed using the flotation and McMaster procedures. The binary logistic regression test was used to examine the odds of the prevalence and confidence interval of each risk factor, and the relationship between the risk factors and protozoan parasites and helminths was examined. The mean differences of the faecal egg count or egg per gram of faeces were also compared using ANOVA. The study’s findings showed that gastrointestinal parasites are common in dogs in the locations under investigation. The overall prevalence of gastrointestinal parasites found in this study was greater than the 19.6%, 33.3%, and 39% prevalences found in earlier studies by [41–43].

However, the overall rate found in this study is generally lower than what has been found by [22–24, 44–47]. These differences in the prevalence of gastrointestinal parasites in dogs may be due to changes in climatic circumstances, animal management, sampling procedures, demographic factors, anthelmintic use, and diagnostic methods used [48, 49].

Compared to mixed infections, single parasite species infections seem to be far more prevalent. This is consistent with previous findings of [24] in Ethiopia’s Hawassa town. Numerous other earlier research have reported similar findings [23, 45]. Conversely, research conducted in different regions of Ethiopia by [21, 50] have shown that the proportion of mixed species infections is significantly higher than that of monoinfection by a single parasite. As previously mentioned, these differences in the parasite population’s makeup could be caused by environmental contamination levels as well as feeding and management practices. The likelihood of finding eggs during faecal analysis may also be decreased by the fact that parasites like some cestode and *Strongyloides* species often pass gravid segments or L1 larvae in faeces instead of eggs [51].

The current study showed that adult dogs had a higher prevalence of gastrointestinal parasites than puppies and young dogs is consistent with data from [13, 22–24, 44, 45, 47, 51]. Adult dogs may have greater opportunities to interact with other dogs and contaminated settings, which is likely the cause [51]. Other earlier findings, however, have demonstrated that infection rates were higher in younger dogs than in adults [46, 50]. This could point to variations in the way the animals are cared for, where consistent use of anthelmintic drugs may lessen positive in mature dogs. However, puppies are susceptible to infection by some common nematode species through congenital and transmammary mechanisms, and the risk of infection may increase until treatment is initiated [46].

This study’s finding that female dogs had a far greater prevalence of gastrointestinal parasites than male dogs is consistent with earlier researches by [4, 10, 22, 52]. Contacts with several male dogs during estrus and the stress of pregnancy and lactation may contribute to the greater prevalence of infection in females by adversely influencing their immune systems [53]. In addition, female dogs are more likely to come into contact with contaminated environments while they are looking for food, mostly to feed their pups [56]. Another explanation is that tissue-encysted *Ancylostoma* and *Toxocara* worms may get reactivated if the females are pregnant, and some of them may develop in the intestines before releasing eggs in the form of faeces. Contrary to this widespread belief, it has been observed that male dogs have a higher frequency of gastrointestinal parasites than female dogs [13, 23, 24, 42, 44, 51].

Dogs with diarrheal faecal consistency had a significantly greater prevalence of gastrointestinal parasites, which may indicate that the animals were affected with gastrointestinal parasitism-related clinical disease. This is consistent with earlier researches shown by [43, 54]. Diarrhoea is one of the primary clinical signs of gastrointestinal parasitism [53], and it may be caused by the helminth parasite’s inflammation and destruction of the absorptive epithelium of the gastrointestinal tract [54].

In the current study, giving dogs raw food was significantly linked to a higher prevalence of gastrointestinal parasites than giving them cooked food on a regular basis. This supports the earlier studies by [4, 43, 55, 56], which found that dogs given raw animal items and leftovers from homes and restaurants had a significantly higher risk of contracting gastrointestinal parasites than dogs fed properly prepared or packaged feed. Dogs fed raw feed had the highest prevalence of gastrointestinal helminths (93.7%), followed by dogs that occasionally fed prepared (37.5%) and raw animal products (90.7%) [20]. The fact that cooking can destroy or inactivate infectious eggs or cysts of gastrointestinal parasites that can be spread among dogs through feed explains why dogs that were fed raw had the highest incidence of these parasites [4].

Despite the fact that every dog examined in this study was owned, over 64% of them fell into the category of stray or outside canines. Compared to dogs housed in a home/compound, dogs that have unrestricted access to the outdoors are much more likely to get gastrointestinal parasite infections. This supports the findings of [10, 47, 51]. Free-roaming pets have a preference for hunting and consume mice, rats, insects, mollusks, poultry, migratory birds, frogs, and small mammals [57]. These foods increase the likelihood that dogs will come into contact with infectious parasite stages on the ground or in intermediate/paratenic hosts. This suggests that food items that are discarded into the environment will be more likely to include soilborne parasites, such as the carnivorous hookworms and the ascarids, which are the most common. Dogs that roam around and scavenge seem to be more likely to come into contact with a contaminated environment [47]. Conversely, the highest incidence (86.3%) was found in dogs that shared a shelter with family [44].

*Toxocara canis* and *Ancylostoma* spp. were frequently found parasites in dogs, which is consistent with study reports from Ethiopia and other countries [22, 24, 41, 45, 47, 58]. These results are readily explained by inadequate hygienic circumstances, the use of anthelmintics, and a lack of health management measures [59].

The dominance of these parasites is expected due to their very high fecundity that allows easy contamination of the environment. In this regard, this study has also demonstrated that *Toxocara* was the most prolific nematode parasite followed by *Physaloptera* and *Ancylostoma* species. The ability of the parasites to pass from generation to generation through transplacental and transmammary routes [60] might have also contributed to the high prevalence. Female *Toxocara* species produces an average of 12500 eggs per day, the range being 9500–55000). Specifically, presence of the free-living dogs and daily discharge of *Toxocara* eggs in the environment (about 200,000 eggs/day) can create a favourable ground for persistence of infection [61].

From cestode parasites, *Dipylidium caninum* eggs were the most prevalent compared to *Taenia* and *Echinococcus* species indicating that infections obtained from the environment were more common than those contracted by eating raw animal items such offals. This agrees with the reports of [41] in Mexico and [24] in Hawassa town while other researchers have reported either lower prevalence [43] or higher prevalences [22, 23, 46, 47, 58]. Such variation could be associated with variation in the diagnostic techniques used, the study population and the agroecological factors affecting the distribution of flea intermediate hosts. For example, *Dipylidium* egg clusters are frequently observed adhered to the perineal region [62], hence sample collection methods may impact the degree of infection detection. *Dipylidium* is a flea-born parasite. Flea larvae are infected by consuming eggs originating from either gravid segments passed in faeces or eggs from degenerated segments within the intestine. Animals get infected through accidental ingestion of infected fleas infesting their body [63]. The limited amount of infected offals consumed may be the reason for the comparatively low prevalence of cestode parasites in this study when compared to the dominant nematodes. Another explanation for the low prevalence would be that the intact gravid segment’s genital pore is closed, which stops free shedding of eggs inside the digestive tract [55].

Despite its extremely low prevalence, *Giardia* was one of the most common protozoan parasites found in dogs. Relatively lower prevalence in Egypt [4] and higher prevalences in Wondo Genet town of Ethiopia [22], Nigeria [46] and Morocco [44] were recorded. Although the zoonotic potential of canine giardiasis remains largely an unresolved issue [2], dogs and cats can carry strains of *Giardia* which are potentially infective to humans, and therefore, the zoonotic potential must be considered, especially for immunocompromised people [64].

Concerning the average egg count of the specific gastrointestinal parasites, the mean egg count was the highest in case of *Toxocara canis* followed by *Physaloptera* spp. and *Dipylidium caninum* with moderate parasitic load. The current finding was higher than the mean count reported by [65]. Whereas [66] reported that *Ancylostoma* spp. and *Uncinaria* spp. had the highest and least egg per gram of faeces (EPG), respectively. This variation could be attributed to the differences in health care and degree of environmental contamination with infective stage, frequent mixing of pets with stray dogs which might have the infections, lack of awareness of dog parasites and their control strategies [65]. Specifically, presence of the free-living dogs and daily discharge of *Toxocara* eggs in the environment (about 200,000 eggs/day) can be considered as a reason of this fact why ascaridoid nematodes are prevalent [61]. The degree of environmental contamination with nematodes and cestodes is also crucial for human health. Considering the information obtained from dog owners, it may be concluded that a high number of EPG is due to occasional and accidental dogs deworming [67].

The current study’s limitation is that, because of budgetary and resource limitations, molecular tests were not used to corroborate these findings; instead, this method was left for future study on the identification of gastrointestinal parasites in dogs.

## 5. Conclusion and recommendations

The total prevalence of gastrointestinal parasites in dogs was found to be high in this study (53.1%). The majority of monoparasite species (28.2%) are nematode parasites. There was a statistically significant higher prevalence of gastrointestinal parasites in female dogs, adults, dogs that were given raw food, dogs kept free outdoor, and dogs from highland areas. Faecal consistency has statistically significant association with the prevalence of gastrointestinal parasites in the examined dogs. *Ancylostoma* spp. was the most common parasite (16%) detected followed by *Dipylidium caninum* (5%) and *Giardia* spp. (3.9%), suggesting that gastrointestinal parasite infections are still common enough to affect dogs in the studied locations, resulting in these animals’ morbidity and mortality. The infection was moderately severe. One health approach is necessary to address this public health hazard because parasitic diseases in dogs can have a substantial negative economic impact in addition to being a serious public health concern.

Given all of this evidence, more thorough research is required to completely comprehend the zoonotic and economic effects of many gastrointestinal parasites in dogs. To identify the parasites to species level in wider study areas in various agro-ecologies and seasons, comprehensive epidemiological and genetic investigations should be carried out. Additionally, it is strongly advised that the general public should be made aware of the prevalence, effects on public health, and financial implications of dog gastrointestinal parasites.

## Supporting information

S1 Data Data-dog GIT parasites-PONE-D-24-49776R1_FTC. (XLSX)
